# A scoping review about the efficacy of clonazepam for the treatment of benign essential blepharospasm

**DOI:** 10.1007/s10072-026-09172-4

**Published:** 2026-06-15

**Authors:** Angelo Torrente, Paolo Alonge, Antonia Pignolo, Eloise Lo Mauro, Roberto Monastero, Angelo Labate

**Affiliations:** Department of Biomedicine, Neuroscience and Advanced Diagnostics, University of Palermo, Palermo, Italy

**Keywords:** Benign essential blepharospasm, Clonazepam, Benzodiazepines, Dystonia

## Abstract

**Background:**

Benign Essential Blepharospasm (BEB) is a focal dystonia that leads to an increased rate of eyelid closure due to involuntary activation of the orbicularis oculi muscles. In severe cases, daily activities can significantly be affected, with reduced quality of life. Injections of OnabotulinumtoxinA are often used as valid therapeutical option. However, this treatment may not be tolerated by all patients, considering its invasive nature and the need for access to specialized centers. Despite oral agents, such as benzodiazepines (particularly clonazepam), are often used as a first-line treatment, evidence regarding their efficacy remains fragmentary.

**Objective:**

This scoping review aims to evaluate the current evidence for the efficacy of clonazepam for benign essential blepharospasm.

**Methods:**

Articles in English about the use of clonazepam in the treatment of BEB were searched for in three major databases (PubMed, Scopus, Cochrane).

**Results:**

The search yielded 259 results. After screening, 13 papers were sought for retrieval. After assessing their suitability, 2 articles were included in the review: one open-label trial and one case report. Despite some limitations, both showed the effectiveness of clonazepam in BEB.

**Conclusion:**

Although some evidence supports the use of clonazepam in the treatment of BEB, high-quality studies are still needed to clarify aspects such as response rates, optimal dose, and other disease management issues. This information may be useful for clinicians who are unable to refer their patients to specialized centers and must rely on their own experience and anecdotal evidence for patient management.

## Introduction

Blepharospasm is a movement disorder under the umbrella of focal dystonias that affects the facial region and causes an increased frequency of eyelid closure due to the involuntary activation of the orbicularis oculi muscles [1]. When the disorder is isolated, it is usually referred to as “benign” and “essential” (i.e., benign essential blepharospasm – BEB). On the other hand, blepharospasm can otherwise manifest in the context of other disorders such as Meige syndrome or other dystonias [2]. BEB pathophysiology is not entirely understood yet and it appears that several factors are involved. Particularly, one of the key factors may be low dopamine activity or dopamine susceptibility at D2 receptors within the basal ganglia [3]. Functional imaging and electrophysiological studies indicate abnormalities in cortical excitability, thalamocortical circuits, and sensorimotor integration, which could explain phenomena such as the “sensory trick” [4]. Moreover, genetic predisposition may also be involved, as some patients show positive family history, and certain genetic variants have even been associated with BEB [5].

BEB has a prevalence of 1.4–13.3 per 100,000 inhabitants, with an incidence of approximately 2,000 new cases per year [2, 6]. Its onset usually occurs between the 5th and 7th decade of life, with a higher prevalence in women [3]. Clinically mild cases show just an increased blink rate, while in more severe forms patients may experience forced eyelid closure leading to functional blindness. The manifestations of BEB may initially be unilateral and involve only one eye, but over time the contractions of the orbicularis oculi become symmetrical and synchronous. The frequency of contractions may be aggravated by environmental factors (e.g., bright lights, smoke or wind), psychological stress, or actions such as up- or down-gaze, reading or looking at a screen [7]. BEB main diagnostic findings include the presence of stereotyped, bilateral, synchronous spams of the orbicularis oculi muscle that cause narrowing or closure of the eyelids, in addition to an effective sensory trick (as per other dystonias), or an increase in the frequency of eyelid blinking at rest [8]. Moreover, the inability to voluntarily suppress spasms is also considered an additional specific diagnostic feature, though less sensitive [9]. Despite being described as “benign”, BEB significantly impairs quality of life, extending beyond motor symptoms. Patients with BEB report severe limitations in daily activities, including difficulty driving and visual function, as well as increased symptoms of depression and anxiety [10].

Regarding the pharmacological management of BEB, one of the most used treatments is botulinum neurotoxin-A (BoNT-A), which induces chemodenervation by blocking the release of acetylcholine at the neuromuscular junction level [11, 12]. BoNT-A is a convenient therapy since it is administered locally and usually shows few side effects that may be related to the spread of the drug beyond the target muscles (e.g., eyelid ptosis) [12]. There is evidence regarding the efficacy and tolerability of repeated injections, even in the long term [13]. Despite the clear advantages of BoNT-A, some patients may not respond to the therapy [14]. Furthermore, BoNT-A treatment requires access to specialized clinics, which may be logistically or financially unfeasible for some patients. Because of the mentioned limitations, evidence regarding alternative therapeutic strategies is therefore needed. Notably, benzodiazepines, and clonazepam in particular, represent the first-line treatment for focal dystonias [15]. However, evidence on the efficacy of clonazepam in BEB is still fragmentary and controversial, so further research is desirable on the topic. Therefore, this scoping review tries to answer the question “what is the evidence for the efficacy of clonazepam for benign essential blepharospasm?”.

## Methods

The review examined all available evidence from original articles written in English concerning the use of clonazepam in the management of BEB. The articles included in the literature search date up to April 27th, 2026. To be included in the review, the articles had to address the management of BEB using clonazepam. The use of clonazepam in the context of a syndrome that includes blepharospasm among other symptoms was not considered for the review. Existing reviews were not considered for the search outcome and could have been used for argumentation purposes only. The following search strategy was used for three of the major bibliographic databases: (i) PubMed (blepharospasm [all fields]) AND (clonazepam [all fields]); (ii) Cochrane blepharospasm: ti, ab, kw AND clonazepam: ti, ab, kw; (iii) Scopus TITLE-ABS-KEY (blepharospasm) AND TITLE-ABS-KEY (clonazepam). Two authors (A.T. and A.P.) examined the title and (if necessary) the abstract of the search output to select relevant articles for full-text review. When the two authors disagreed about the inclusion of an article, a third senior author (R.M.) made the final decision.

## Results

### Databases output

The PubMed search yielded 23 articles, 8 of which contained direct information relevant to the research question, including 1 review, 1 open-label trial, 3 case series, and 3 case reports. The Cochrane search yielded only one duplicate result, namely 1 clinical trial. The search in the Scopus database yielded 235 documents, of which 11 papers contained direct information for the research question: 2 retrospective studies (1 duplicate), 1 prospective article (Japanese), 1 case report (duplicate), 5 reviews (1 Japanese), 2 book chapters.

### Search outcome

**Fig. 1:**
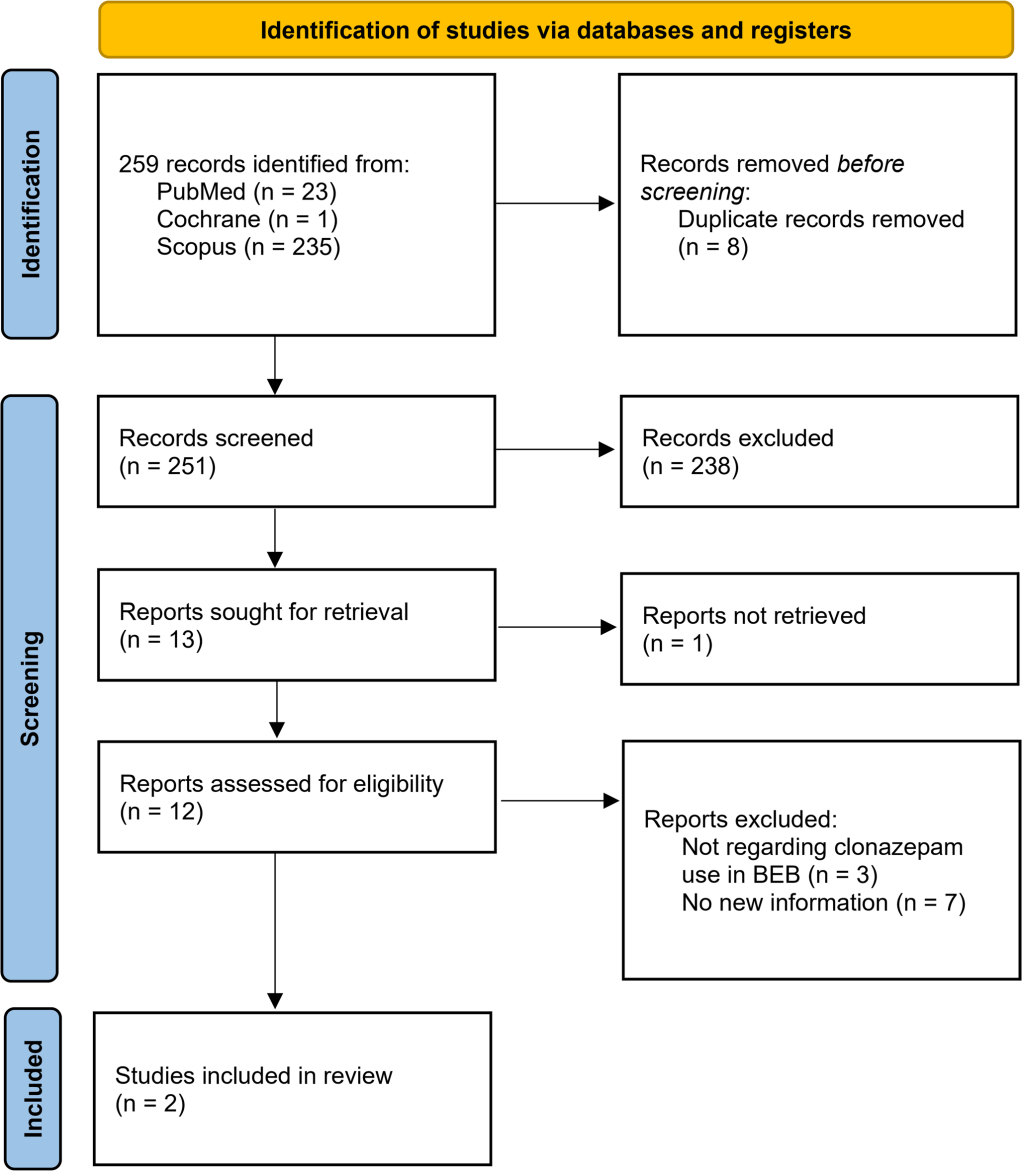
PRISMA flow diagram summarizing the search output and review

The search yielded 259 results, including 8 duplicates. After discarding non-pertinent records (from the selection of titles and abstracts) and papers not written in English, 13 papers with full text were evaluated. After applying the inclusion and exclusion criteria, 11 papers were discarded (3 did not contain direct information on the use of clonazepam in BEB, 5 were reviews and 2 were book chapters that did not contain new information from the literature, 1 article presenting just the abstract with full text not available), leaving only 2 articles to be included in this review. For further information, see the PRISMA flow diagram below (Fig. 1).

The main characteristics of the two studies included in the review are shown in Table 1.

**Table 1:** Main characteristics of the studies included in the review

Reference	Patient group and intervention	Study type	Outcome	Key results	Study weakness
Woo et al., 2020 [16]	41, clonazepam 1.5 mg per day for 1 week	Open label	Subjective treatment response (beneficial/not beneficial)	25% beneficial	No quantitative measure
Ballard, 1989 [17]	1, clonazepam 1 mg bid titrated to 1.5 mg bid	Case report	Number of blepharospasm episodes per day	Positive response	Single case report

Woo et al. presented a study involving patients affected by various types of dystonias, including BEB, treated with different oral medications (clonazepam, trihexyphenidyl, nortriptyline, baclofen, levodopa). Patients underwent separate, consecutive 7-day trials of different oral agents (1.5 mg of clonazepam, 6 mg of trihexyphenidyl, 30 mg of nortriptyline, 30 mg of baclofen, and 150 mg of levodopa per day) and were asked to report whether they experienced a beneficial or non-beneficial response. Among the 172 patients included in the study, 41 were affected by blepharospasm. Interestingly, clonazepam demonstrated the highest subjective efficacy within this subgroup, with 25% of patients reporting clinical improvement, despite the relatively low dose and the very short treatment period studied. These findings, although limited, suggest a potential role for clonazepam as an alternative or adjunctive oral therapy in patients with BEB, particularly when more established interventions are not accessible or feasible.

In contrast, Ballard provided anecdotal but compelling evidence through a single case report of a patient who developed severe blepharospasm symptoms associated with schizophrenia. The use of neuroleptic drugs is not mentioned, but the author report that the patient’s psychiatric condition was stable, functioning well in the community with a normal quality of life until he developed blepharospasm. The patient’s disabling eyelid spasms, which interfered with his daily activities, responded remarkably well to clonazepam titrated to 1.5 mg twice daily, with almost complete resolution of symptoms. This observation reinforces the hypothesis that clonazepam may exert a clinically meaningful effect on BEB in selected patients, and that dose titration over time may be essential for achieving therapeutic efficacy.

## Discussion

The treatment of BEB is a topic that has not been explored in depth in scientific literature, as research has produced just a few results. The only study involving multiple patients that met the search criteria showed data on patients affected by blepharospasm treated with various oral medications; among them, 41 were treated with clonazepam and the patients’ subjective response to 1-week therapy was evaluated [16]. Although the response rate may seem moderately low, it was the highest among all the medications investigated. Furthermore, the authors studied only a low initial dose for a limited time; perhaps a higher dose or longer therapy could have shown better results. The only other article that met the search criteria was a case report describing a very positive response to oral therapy [17]. However, both the analyzed articles have some limitations: first, the lack of a standardized outcome measure, such as a validated instrument to quantify blepharospasm severity or its improvement (the open-label study only assessed patients’ subjective response); second, the absence of placebo-controlled conditions and insufficient follow-up duration; third, the second article was the description of a single case report, with limited information regarding the chronic use of any other medications. Nevertheless, the patient’s psychiatric condition was stable and well-balanced, so it is reasonable to infer that blepharospasm arose as an independent condition (BEB).

For the study of blepharospasm, it is advisable to use a recognized scale for the identification and monitoring of symptoms, such as the Burke–Fahn–Marsden Scale, the Global Dystonia Severity Rating Scale, the Jankovic Rating Scale, or the Blepharospasm Severity Rating Scale [18]. In addition to objective examination, clinicians should even consider patients’ subjective impact, using, for example, the Blepharospasm Disability Index [19]. Such instruments would increase the reliability of results in the research settings.

The few other relevant studies or reports found in literature were not included in the review as they did not concern BEB but included patients with syndromic blepharospasm or blepharospasm associated with other dystonias. Nevertheless, evidence regarding the efficacy of clonazepam in other forms of blepharospasm was reported [20–23]. Thus, it is possible to suppose that this oral drug would be effective even in BEB, considering that syndromic dystonias are generally more difficult to treat.

The rationale to use clonazepam in dystonias reflects its effect on increasing GABAergic inhibitory action at the basal ganglia level [24]. This inhibition of the basal ganglia circuitry reduces the high blinking frequency associated with blepharospasm [20, 25]. Although benzodiazepines may be associated with some side effects such as drowsiness, clonazepam shows a good tolerability profile and efficacy in BEB, so it could be considered as a convenient and easily manageable therapy [26, 27].

The limited number of studies on this topic may depend on several factors: for instance, there are other therapies for BEB, such as onabotulinumtoxinA, which is effective and shows a good tolerability profile. However, some patients may refuse the idea of injectable therapy or may have limited access to centers where injectable therapy with onabotulinumtoxinA is performed. In such situations, oral treatments may represent the best choice. On the other hand, neurologists working in small centers may not have onabotulinumtoxinA therapy available and may use clonazepam for BEB, but they may not be interested in publishing the results of their work. Conversely, in larger university settings, injectable therapies may be used more frequently, and researchers may be more inclined to publish these results and less those ones related to clonazepam. Consequently, the impact of clonazepam on BEB is still poorly described. Given the relatively high incidence of BEB and the disability associated with the disease, a better definition of treatment options is essential to improve its management. This is particularly important for patients who do not have access to specialized centers and must consult general neurologists. In the absence of high-quality evidence, these physicians must rely on personal experience or anecdotal evidence to evaluate treatment options, titrate doses, predict outcomes, and address other management issues.

## Conclusion

The data in this review do not provide statistical evidence to support the use of clonazepam in the treatment of BEB. One open-label study suggests that a quarter of patients may respond favorably after one week of treatment with this drug, while another case report suggests its effectiveness on symptoms. Although there is some evidence relating to other forms of blepharospasm, there are no specific and high-quality studies on BEB. Ultimately, although BoNT-A remains the first-line therapy for BEB due to its proven efficacy and safety profile, clonazepam may represent a useful oral option in specific settings, such as limited access to specialized injection centers, patient refusal of injectable treatments, or as an adjunctive strategy in refractory cases. Given the relatively high prevalence of BEB and its significant impact on patients’ quality of life, adequate randomized controlled trials are needed to confirm the efficacy of clonazepam as an alternative or complementary treatment which may be chosen in various situations in clinical practice.
